# Disruption of Spatial Task Performance in Anorexia Nervosa

**DOI:** 10.1371/journal.pone.0054928

**Published:** 2013-01-18

**Authors:** Dewi Guardia, Aurélie Carey, Olivier Cottencin, Pierre Thomas, Marion Luyat

**Affiliations:** 1 Laboratoire de Neurosciences Fonctionnelles et Pathologies, EA-4559, Université Lille Nord de France, Lille, France; 2 Service d'Addictologie, Hôpital Calmette, CHRU de Lille, Lille, France; 3 Service de Psychiatrie, Hôpital Fontan, CHRU de Lille, Lille, France; 4 UFR de Psychologie, Université Lille 3, Villeneuve d'Ascq, France; University of Bologna, Italy

## Abstract

In anorexia nervosa (AN), body distortions have been associated with parietal cortex (PC) dysfunction. The PC is the anatomical substrate for a supramodal reference framework involved in spatial orientation constancy. Here, we sought to evaluate spatial orientation constancy and the perception of body orientation in AN patients. In the present study, we investigated the effect of passive lateral body inclination on the visual and tactile subjective vertical (SV) and body Z-axis in 25 AN patients and 25 healthy controls. Subjects performed visual- and tactile-spatial judgments of axis orientations in an upright position and tilted 90° clockwise or counterclockwise. We observed a significant deviation of the tactile and visual SV towards the body (an A-effect) under tilted conditions, suggesting a multisensory impairment in spatial orientation. Deviation of the Z-axis in the direction of the tilt was also observed in the AN group. The greater A-effect in AN patients may reflect reduced interoceptive awareness and thus inadequate consideration of gravitational inflow. Furthermore, marked body weight loss could decrease the somatosensory inputs required for spatial orientation. Our study results suggest that spatial references are impaired in AN. This may be due to particular integration of visual, tactile and gravitational information (e.g. vestibular and proprioceptive cues) in the PC.

## Introduction

Key symptoms of anorexia nervosa (AN) include (i) disturbance in the way in which one's body weight or shape is experienced, (ii) an undue influence of body weight or shape on self-evaluation and (iii) a persistent lack of recognition of the seriousness of low body weight; in fact, AN patients perceive themselves to be larger than they really are [1]. This alteration in body perception may relate to various levels of representation, such as the body schema and the body image [2]. The body schema is a dynamic, unconscious, sensorimotor representation of the body that is built on the basis of tactile, kinesthetic, visual and labyrinthine inputs. It is elicited by action, regardless of whether the latter is imagined, anticipated and/or executed [3]–[5]. The notion of body image is more complex and concerns not only perceptual representations of the body but also semantic, aesthetic and emotional aspects that are not used for action *per se* [2], [4].

Most of the studies in AN to date have focused on cognitive and emotional aspects of body image [6]–[9]. However, some authors have suggested that the body schema may also be affected [10]–[14] as a result of dysfunction of the parietal cortex (PC) in general and the right superior parietal lobule in particular [13], [15]–[16]. The latter structure was found to be crucial for establishing a coherent body schema [17]. However, the development of a coherent representation of the body requires the prior integration and synthesis of visual, tactile, vestibular and proprioceptive information. Even though the exact interpretations differ, several researchers have evidenced a disturbance of multisensory integration in AN [18]–[24]. For instance, Case *et al.* [23] used a size-weight illusion (SWI) paradigm to demonstrate the presence of impaired visuoproprioceptive integration in AN. A SWI arises when two objects of equal weight but different sizes are weighed [25], with participants consistently under-estimating the weight of the larger of the two objects. Even though several explanatory hypothesis exist, it is generally assumed that the SWI is due to a conflict when integrating visual information (in everyday life, large objects are usually heavier than small objects) and tactile perceptions (the two objects have the same weight). Hence, Case et al. [23] found that the SWI was less intense in AN and suggested that the patients could be less sensitive to visual information and more sensitive to proprioceptive inputs. Indeed, as demonstrated by Keizer et al. [22], preferential weighting of proprioceptive information and overestimation of the tactile body image would disturb the body schema. Moreover, according to Pollatos and colleagues [21], patients with AN may present low interoceptive awareness. This altered perception of the body interoceptive signals may also be involved in the development of an altered body schema. However, AN patients experience the rubber hand illusion (RHI) more strongly than healthy controls do [24]. In the RHI [26], participants view a fake hand being stroked with a paintbrush. At the same time, the experimenter applies identical brushstrokes to the participant's own hand, which is out of the participant's view. If these items of visual and tactile information are applied synchronously and if the fake hand's visual appearance and position are similar to those of the participant's own hand, then some people may feel that the stimuli are coming from the dummy hand and even that the latter is, in some way, part of their own body. This phenomenon requires multisensory integration and the dominance of visual information on hand location over proprioceptive information. Although the AN patients' greater sensitivity to visual information in this task somewhat contradicts their greater sensitivity to proprioceptive information in the SWI paradigm found by Case et al. [23], these results generally suggest that multisensory integration in AN is different from healthy subjects. The latter may be related to overestimation of the body schema.

Spatial cognition corresponds to understanding and conceptualizing visual representations and spatial relationships in learning and performing a task. Research evidence suggests that the parietal lobes are extensively involved in spatial analysis, including the analysis of location and spatial relationships. Even though the PC is viewed as the main locus of the body schema, this structure is involved in many other features requiring multisensory integration [27]. For instance, the PC is thought to sustain the emergence of a supramodal reference frame involved in spatial orientation constancy [28], [29]. Spatial orientation constancy is defined as the central nervous system's capability to maintain the sense of gravitational, vertical orientation (i.e. the sense of verticality) despite inclination of the body (i.e. the egocentric reference frame) and/or the visual reference frame [30]. Hence, when we tilt our body, our perception of the world remains the same. Indeed, the integration of tactile, proprioceptive, visual and vestibular information is required for the development of an allocentric reference frame that enables spatial orientation constancy [31]–[33]. The adjustment of a bar to match the subjective vertical (SV) is a frequently used, simple and effective method of measuring spatial orientation constancy [34].

In fact, spatial orientation constancy is far from constant in healthy subjects under some circumstances. In the dark, head and/or body tilts cause slight but systematic deviations of the SV [30], [32]–[33]. Whereas A-effects (deviations of the SV towards the head's axis) are observed in vision and with large tilts, E-effects (deviations of the SV away from the head's axis) are usually seen with tactile adjustments [31], [32], [35], [36]. It must be noticed that if the E-effect is typically observed in tactile modality in healthy subjects, some researchers have found either a slight A-effect [37], or no significant effect [29] of head tilt. Funk and colleagues [29] recently investigated the effect of passive lateral inclination of the head on the visual SV, the tactile SV and the tactile horizontal in (i) neglect patients, (ii) control patients with left- or right-sided brain damage but not neglect and (iii) healthy controls. Neglect patients consistently displayed an A-effect in both the visual and tactile modalities. This might have been caused by abnormal attraction of the SV by the idiotropic vector [38], on the basis of the head's actual orientation [29]. Greater weighting towards the head's egocentric reference frame could be interpreted as the consequence of impaired processing of vestibular information in neglect patients. Given the parallels between neglect subjects and anorexia patients reported in the literature [13], [39], we performed a preliminary study of tactile SV perception in AN and healthy controls [37] by investigating the effect of passive lateral whole body inclination on the tactile SV. For body-tilted conditions, we observed an increased A-effect in AN patients. This effect was similar to that found by Funk and colleagues [29] in neglect patients and might be due to higher weighting of the egocentric frame of reference. However, in order to perceive the SV, the participant must first compute an angle between the rod line and the body Z-axis (i.e. the head-to-foot axis) according to the perceived body orientation [40]. In this sense, the perception of SV is not a direct measure of the involvement of the egocentric reference frame. A measure of the participant's perception of the body axis in space is probably a more direct index of the body schema. Moreover, our previous study only explored the tactile SV and the data obtained could resulted from a specific tactile impairment in AN, as has been showed by several authors. For instance, Grunwald *et al.* [16] asked participants had to manually adjust a bar (in the absence of visual feedback) into a parallel position, relative to a reference bar sensed by the other hand. The patients with AN had trouble copying the angles via haptic perception. In a previous study, Grunwald *et al.* [10] assessed a tactile exploration task that consisted in palpating the structure of different reliefs with the eyes closed. Next, the participant had to reproduce each structure on a piece of paper. The quality of the reproductions made by AN patients was notably worse than that observed in control subjects. Reproduction quality was still impaired after weight gain. Tchanturia *et al.* [41] found that patients with AN had impairment performance in the haptic illusion task, during which the subject has to use tactile information to discriminate between objects of different sizes and textures. In view of these deficits in tactile/haptic modalities, we considered that it was essential to determine whether the effect that we had found previously (i.e. a greater A-effect in a tactile modality) was also present in a visual modality.

Hence, in the present study, we used two different modalities to evaluate spatial orientation constancy and the perception of body posture in AN patients. We investigated the effect of passive lateral body inclination on (i) the visual and tactile SV and (ii) perception of body Z-axis in AN patients and healthy controls. Subjects performed visual- and tactile-spatial judgments of these orientations in an upright position and with lateral whole-body tilt (90° clockwise or counterclockwise from the vertical). Given the PC's involvement in multisensory integration and spatial orientation constancy, we expected to find disturbances of spatial orientation constancy in AN patients with, notably, greater A-effects for the SV in both visual and tactile modalities. We added two additional tasks, in order to check that the AN patients' ability to achieve tactile and visual discrimination was intact.

## Methods

### Ethics statement

This study was approved by an institutional review board (*Comité de Protection des Personnes Nord Ouest IV*; study number 2007-A01413-50). The study complied with the tenets of the Declaration of Helsinki. Each participant received a study information sheet and provided her prior, written, informed consent to participation. For participants under the age of 18, parental consent was also required.

### Participants

The present study included 50 young female adults: 25 AN patients and 25 healthy controls matched for age and educational level. All AN patients were recruited from an eating disorder clinic. The clinical evaluation of the participants by the psychiatrist did not reveal any perceptual, attentional or intellectual impairment. The AN patients met the DSM IV-TR criteria for the condition [1]. Patients with the following comorbidities were excluded from the study: bulimia, anxiety disorders, mood disorders, psychotic disorders and substance abuse. All AN patients belonged to the restrictive subtype of AN. The mean duration of illness was equal to 4.57 years (SD =  6.52 yrs; Min-Max =  1–28,5 yrs; Median  = 2.67 yrs).

Healthy controls were recruited from a college and university population. The Mini-International Neuropsychiatric Interview (conducted by a psychiatrist) confirmed the absence of comorbidities in both groups, according to the DSM IV criteria [42]. All controls had a healthy body mass index (BMI: weight/height^2^; mean = 21.53; SD = 1.8; median =  21.35; range: 18.55–24.56 kg/m^2^). We chose not to recruit male participants for the present study, given their low number and high rate of psychiatric comorbidities in the AN population. Participants with a history of neurological or vestibular problems or those taking psychotropic medication at the time of the study were also excluded.

### Materials and procedure

#### Morphological and clinical parameters

The experimenter's assessment of height and weight was standardized. Analysis of handedness was performed using the Edinburgh Inventory [43]. Body dissatisfaction and concerns about weight and shape were assessed in both control and AN groups with the Eating Disorder inventory-2 (EDI-2). The EDI-2 scores 11 psychological features commonly associated with eating disorders [44]. The total EDI-2 score and the “interoceptive awareness” subscore were used in the present study.

#### Discrimination tests

The discrimination tests were used to check the efficiency of the visual and tactile modalities in other tasks than spatial orientation tasks. Two experimental conditions were tested.

Under the tactile condition, the participant was blindfolded with a mask. The stimuli consisted of 10 cubes (with a side length ranging from 1 cm to 10 cm) that the participant could manipulate freely. Each trial consisted of the presentation of two stimuli consecutively: a standard stimulus (the 5 cm cube) and a variable stimulus (one of the cubes with a side length of between 1 cm and 10 cm). At each trial, the participant had to explore the each shape of the two presented cubes with both hands and had to judge whether the variable stimulus was smaller than the standard stimulus, equal in size to the standard stimulus or greater than the standard stimulus. Each response was made verbally and noted by the experimenter on a record sheet. In accordance with the constant stimulus method, each variable stimulus was presented three times in random order.

Under the visual condition, the stimuli consisted of 10 different squares with a side length ranging from 1 cm to 10 cm. At each trial, the standard square (side length: 5 cm) and the variable stimulus were presented consecutively on a 14-inch computer monitor (interstimulus duration: 500 ms). In accordance with the constant stimulus method, each pair of stimuli was presented three times in random order using E-prime software (Psychology Software Tools, Sharpsburg, PA, USA). The participant was instructed to judge whether each variable stimulus was smaller than the standard stimulus, equal in size to the standard stimulus or greater than the standard stimulus by pressing the corresponding key on a keyboard. There was no limit on the participant's response time. We recorded the number of incorrect judgments under each condition.

#### Spatial orientation tests

Body Z-axis and SV tasks were studied under tactile and visual conditions. Under the tactile condition, the material consisted of a rod (length: 20 cm) pivoting around an axis on a circular metal plate (diameter: 30 cm). The rod was connected to a potentiometer which measured the angle (in degrees) from the gravitational vertical with a sensitivity of ±1%. Three postural conditions were tested: sitting upright (0°), body roll-tilted to the left (−90°) and body roll-tilted to the right (+90°). During body roll-tilted tasks, the participant lay on her side. In the SV task, the blindfolded participant was instructed to manually adjust the rod to the gravitational vertical (0°) with either the right or the left hand. The task was performed under each of the three postural conditions (tilt: 0°, −90° or +90°). Six trials were performed for each of the six experimental conditions. In the body Z-axis task, the blindfolded participant was instructed to manually adjust the rod to her body midline. The rod's initial position was alternately +45° or −45° from the Z-axis. Likewise, the twelve experimental conditions were presented to the participants in pseudorandom order. For each trial, the absolute deviation from the gravitational vertical or from the body midline was noted. By convention, deviations to the participant's left (i.e. the rod turned counter-clockwise from 0°) were counted as negative and deviations to the right (i.e. clockwise rotations) were counted as positive. In order to determine the value of the tactile SV and body Z-axis under each postural condition, we computed the mean error (in degrees) over the six trials. In order to compare the precision of the adjustment in the two groups, the individual standard deviation (SD) was also computed over the six trials and under each experimental condition.

For the visual modality, the material was composed of a tunnel (60 cm long) within a metal and plastic frame, the height of which could be adjusted to suit the seated participant. At the bottom of this device was a rotatable metal disc (diameter: 44 cm) bearing a binocularly viewed phosphorescent line (23 cm ×1 cm; visual angle: 21.70°). The back of the disc was graduated in degrees and the display's sensitivity threshold was ±0.5°. The participant had to hold her head against the aperture of the tunnel at four points (both temples, the chin and the top of the head) when sitting upright (0°) and at three points (one temple, the chin and the top head) when body-roll-tilted to the left (−90°) or to the right (+90°). During the experiment, only the phosphorescent line was visible. As for the tactile task, the same three postural conditions were tested: sitting upright (0°), body-roll-tilted to the left (−90°) and body-roll-tilted to the right (+90°). Six trials were performed under each condition. Again, two tasks were tested. In the SV task, the participant was instructed to manually adjust the phosphorescent line to the gravitational vertical (0°) with either the right or the left hand but in the absence of other visual information. The phosphorescent line's initial position was alternately +45° and −45° from the vertical. In the body Z-axis task, the participant was instructed to manually adjust the phosphorescent line to the body midline. The phosphorescent line's initial position was alternately +45° and −45° from the Z-axis. The twelve experimental conditions were presented to the participants in pseudorandom order. For each trial, the algebraic deviation from the gravitational vertical or the body midline was noted. By convention, deviations to the participant's left (i.e. the phosphorescent line turned counter-clockwise from the Z-axis) were counted as negative and deviations to the right (i.e. clockwise rotations) were counted as positive. In order to determine the value of the visual SV and body Z-axis under each postural condition, the mean error (in degrees) over the six trials was computed. In order to compare the precision of the adjustment in the two groups, the individual SD was also computed over the six trials under each experimental condition.

### Statistical analysis

All analyses were performed with Statistica software (version 7.1, Statsoft Inc., Tulsa, OK, USA). Demographic and clinical data with non-normal distributions and non-homogenous intergroup variances were studied with non-parametric Mann–Whitney and Spearman tests. In spatial orientation tests, the respective influences of *group*, *body orientation* and *hand* used were studied in an analysis of variance (ANOVA). An analysis of covariance (ANCOVA) was used to explore the influence of physiological and psychological measures on spatial tasks. The validity of the tests' conditions of application (i.e. normality and homogeneity of variances) had been demonstrated previously.

## Results

### Participants' characteristics

Demographic and clinical data are reported in Table 1. As expected (in view of the matching criteria), there were no significant differences between the two groups in terms of age (mean_AN_ = 22.24 years vs. mean_C_ = 22.88 years; t_48 = _−0.437, p = 0.664), educational level (secondary education: 16 AN patients vs. 20 control participants; university education: 9 AN patients vs. 5 control participants; t_48 = _−1.162, p = 0.251), handedness (right-handed: 20 AN patients vs. 21 control participants; left-handed: 5 AN patients vs. 4 control participants; t_48_ = 0.696, p = 0.489) or height (median_AN_ = 1.665 m and median_C_ = 1.69 m (U = 226.5, Z = 1.268, p = 0.204)). Bodyweight was significantly lower in the AN group (median_AN_ = 42 kg and median_C_ = 60 kg; U = 0.5, Z = −6.054, p<0.001), as well as BMI (median_AN_ = 14.93 kg/m^2^ and median_C_ = 21.35 kg/m^2^; U = 0.5, Z = −6.063, p<0.001). The overall EDI-2 score was significantly higher in the patient group than in the control group (median_AN_: 95, median_C_: 28; U = 44, Z = −4.64, p<0.001), as was the “interoceptive awareness” subscore (median_AN_: 11, median_C_: 2; U = 55.5, Z = −4.37, p<0.001).

**Table 1:** Demographic and clinical data for AN patients and healthy controls.

	AN patients (n = 25)		Healthy controls (n = 25)		
	Mean (SD)	Median (Range)	Mean (SD)	Median (Range)	P-value
Age (years)	22.24 (8.599)	20.5 (15–48)	22.88 (3.632)	24 (16–29)	0.664
Weight (kg)	40.396 (4.932)	42 (29.1–49.6)	60.854 (5.74)	60 (38.5–78	<0.001
Height (m)	1.645 (0.069)	1.665 (1.49–1.75)	1.677 (0.064)	1.69 (1.53–1.8)	0.204
BMI (kg/m^2^)	14.895 (1.107)	15 (12.62–17.57)	21.65 (1.729)	21.3 (18.67–24.56)	<0.001
EDI-2 score					
Total score	95.864 (43.975)	95 (23–205)	31.478 (15.385)	29 (10–135)	<0.001
Interoceptive awareness	11.545 (2.304)	11 (0–24)	2.304 (2.619)	2 (0–17)	<0.001

*BMI: body mass index; EDI-2: Eating Disorder Inventory-2*.

### Behavioural data

#### Discrimination tests

Under the tactile condition, there was no significant difference in discrimination between the groups (U = 299, Z = −0.489, p = 0.624), which showed similar error rates (median_AN_: 3 vs. median_C_: 3). This was also true for the visual modality (median_AN_: 3 vs. median_C_: 2.5; U = 170.5, Z = −0.278, p = 0.781). Hence, performance levels for visual and tactile discrimination of simple shapes (squares of different sizes for the visual condition and cubes of different sizes for the tactile condition) were not different in the two groups.

#### Effects of body orientation on spatial orientation judgments

**Figure 1:**
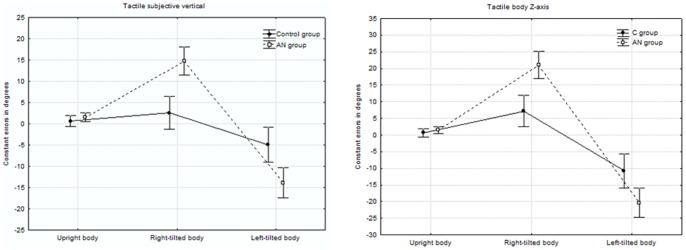
Constant error in degrees in tactile-spatial orientation tasks in AN patients and healthy controls.

The results of the tactile SV task are summarized in Table 2 and Figure 1. An ANOVA of the mean algebraic errors was performed, with repeated measures on both *body orientation* and *hand* and with *group* as a categorical predictor. The analysis revealed a significant effect of *body orientation* (F_(2,92)_ = 53.554, p<0.001). When compared with the upright position (mean_0°_±SD = 1.473°±2.236), the SV in tilted positions deviated towards the body Z-axis (mean_−90°_ = −8.306°±8.81 and mean_+90°_ = 8.012°±9.366). This analysis revealed also a significant effect of *group* (F_(1,46)_ = 4.429, p = 0.041), with a large A-effect and a greater deviation towards the body Z-axis in the AN group. The interaction between *group* and *body orientation* was also statistically significant (F_(2,92) = _18.056, p<0.001). In the upright position, the difference between the two groups' respective performances was not significant (F_0°(1,46) = _2.397, p = 0.128). In contrast, the deviations of the SV towards the body Z-axis (in both left- and right-tilted positions) were significantly more pronounced in AN patients (F_−90°(1,46) = _9.11, P = 0.004 and F_+90°(1,46) = _25.43, p<0.001, respectively). The analysis did not reveal any other significant factors or interactions. Moreover, a similar ANOVA performed on the intraindividual precision of the adjustments (the individual SD) revealed neither a significant effect of *group* nor an interaction between *group* and the other factors (all p >0.1).

**Table 2:** Deviation of the tactile subjective vertical and body Z-axis under the three different body orientation conditions in AN patients and healthy controls.

	AN patients (n = 25)	Healthy controls (n = 25)
	Mean (SD)	Mean (SD
Subjective vertical		
Upright body (0°)		
* Right hand*	0.455 (3.568)	0.04 (2.146)
* Left hand*	2.654 (2.835)	2.567 (3.243)
Right-tilted body (+90°)		
* Right hand*	15.468 (10.601)	2.887 (5.698)
* Left hand*	12.635 (8.187)	0.52 (7.219)
Left-tilted body (−90°)		
* Right hand*	−9.756 (7.009)	−2.713 (8.3)
* Left hand*	−15.57 (11.687)	−4.433 (7.662)
Z-axis		
Upright body (0°)		
* Right hand*	0.427 (3.639)	0.145 (2.162)
* Left hand*	2.86 (2.687)	2.715 (3.224)
Right-tilted body (+90°)		
* Right hand*	20.086 (8.811)	8.083 (8.59)
* Left hand*	16.756 (8.151)	6.647 (8.186)
Left-tilted body (−90°)		
* Right hand*	−14.41 (9.955)	−7 (8.887)
* Left hand*	−23.423 (13.136)	−10.953 (7.319)

*Mean constant error and standard deviation in degrees*.

The results of the tactile subjective body Z-axis task are summarized in Table 2 and Figure 1. The ANOVA with repeated measures on both *body orientation* and *hand* (with *group* as a categorical predictor) again revealed a significant effect of *body orientation*: F_(2,92) = _99.413, p<0.001. When compared with the upright position (mean_0°_ = 1.537°±2.928), the body Z-axis judgments were overestimated under tilted conditions (mean_−90°_ = −14.077°±10.576 and mean_+90° = _14.345°±10.591). The interaction between *group* and *body orientation* was also statistically significant (F_(2,92)_ = 15.815, p<0.001). The difference between the groups' respective performances was not significant in the vertical posture (F_0°(1,46) = _2.397, p = 0.128). In contrast, the deviations of the body Z-axis adjustments in tilted posture were more pronounced in AN patients (F_−90°(1,46)_ = 8.285, p = 0.006 and F_+90°(1,46) = _20.351, p<0.001, respectively). The analysis did not reveal any other significant factors or interactions.

**Figure 2:**
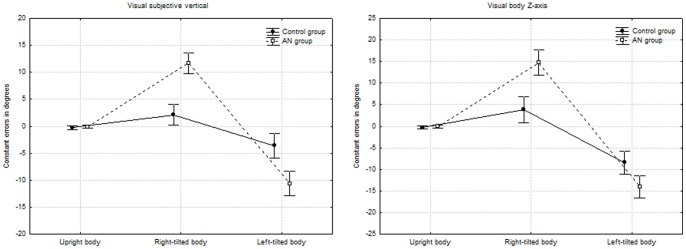
Constant error in degrees in visual-spatial orientation tasks in AN patients and healthy controls.

The results of the visual SV task are summarized in Table 3 and Figure 2. The ANOVA with repeated measures on both *body orientation* and *hand* (with *group* as a categorical predictor) again revealed a significant effect of *body orientation*: F_(2,92)_ = 118.098, p<0.001. When compared with the upright position (mean_0°_ = −0.168°±0.745), the SV in tilted positions deviated towards the body axis (mean_−90°_ = −7.023°±6.601 and mean_+90° = _6.86°±6.835). The interaction between *group* and *body orientation* was also statistically significant (F_(2,92)_ = 9.906, p<0.001). In the upright position, the difference between the groups' respective performances was not significant (F_0°(1,46) = _1.276, p = 0.264). In contrast, the deviations of the SV towards the body axis in both left- and right-tilted positions were significantly more pronounced in AN patients (F_−90°(1,46) = _3.842, p = 0.098 and F_+90°(1,46) = _14.787, p<0.001, respectively). Lastly, the interaction between *handedness* and *body orientation* was also statistically significant (F_(2,92)_ = 8.05, p<0.001). In contrast to the vertical posture, an univariate analysis showed an effect of *hand* when the body was tilted (F_0°(1,46)_ = 2.606, p = 0.113; F_−90°(1,46) = _6.577, p = 0.014; F_+90°(1,46) = _6.813, p = 0.012). When compared with the upright position (right hand: mean_0°_ = −0.108°±0.701; left hand: mean_0°_ = −0.232°±1.099), performance was worse with the hand that was contralateral to the tilt, regardless of whether the body was tilted to the left (right hand: mean_−90°_ = −7.173°±8.08; left hand: mean_−90°_ = −7.179°±6.226) or the right (right hand: mean_+90°_ = 7.444°±7.822; left hand: mean_+90°_ = 6.601°±6.889). No other significant factors or interactions (notably between *handedness*, *group* and *body orientation*) were found.

**Table 3:** Deviation of visual subjective vertical and body Z-axis under the three different body orientation conditions in AN patients and healthy controls.

	AN patients (n = 25)	Healthy controls (n = 25)
	Mean (SD)	Mean (SD
Subjective vertical		
Upright body (0°)		
* Right hand*	0.096 (0.638)	−0.32 (0.712)
* Left hand*	−0.237 (1.251)	−0.227 (0.941)
Right-tilted body (+90°)		
* Right hand*	14.769 (6.266)	3.89 (8.712)
* Left hand*	11.032 (5.545)	1.993 (4.846)
Left-tilted body (−90°)		
* Right hand*	−14.032 (4.785)	−8.43 (7.85)
* Left hand*	−9.955 (4.148)	−4.293 (6.768)
Z-axis		
Upright body (0°)		
* Right hand*	0.1 (0.651)	−0.257 (0.652)
* Left hand*	−0.227 (1.275)	−0.104 (0.73)
Right-tilted body (+90°)		
* Right hand*	15.423 (6.999)	3.36 (9.296)
* Left hand*	11.734 (5.339)	2.123 (4.411)
Left-tilted body (−90°)		
* Right hand*	−13.878 (4.941)	−8.047 (7.947)
* Left hand*	−10.615 (4.425)	−3.6 (6.703)

*Mean constant error and standard deviation in degrees*.

The results of the visual subjective body Z-axis task are summarized in Table 3 and Figure 2. The ANOVA with repeated measures on both *body orientation* and *hand* (with *group* as a categorical predictor) again revealed a significant effect of *body orientation* (F_(2,92)_ = 85.902, p<0.001). When compared with the upright position (mean_0°_±SD = −0.122°±0.923), the body Z-axis was overestimated in the tilted positions (mean_−90°_ = −11.085°±6.923 and mean_+90° = _9.158°±9.164). The interaction between *group* and *body orientation* was also statistically significant (F_(2,92)_ = 22.672, p<0.001). In the upright position, the difference between the groups' performances was not significant [F_0°_(1,46) = 1.276, P = 0.264]. In contrast, the deviations of the body Z-axis in both left- and right-tilted positions were significantly more pronounced in AN patients (F_−90°(1,46) = _15.3, p<0.001 and F_+90°(1,46) = _19.474, p<0.001, respectively). The interaction between *handedness* and *body orientation* was also statistically significant (F_(2,92)_ = 8.632, p<0.001). An univariate analysis showed an effect of *hand* only when the body was tilted (F_0°(1,46) = _2.606, p = 0.113; F_−90°(1,46) = _7.617, p = 0.008; F_+90°(1,46) = _5.436, p = 0.024). When compared with the upright position (right hand: mean_0°_ = −0.078°±0.669; left hand: mean_0°_ = −0.165°±1.036), performance was worse with the left hand, regardless of whether the body was tilted to the left (right hand :mean_−90°_ = −11.019°±7.155; left hand: mean_−90°_ = −11.552°±7.332) or the right (right hand: mean_+90°_ = 9.509°±10.152; left hand: mean_+90°_ = 9.363°±8.938). Lastly, no interactions between *handedness*, *group* and *body orientation* were found (n.s., p>0.1).

#### Effect of the perceptive modality on judgments of spatial orientation

An ANOVA on the mean algebraic errors was performed, with repeated measures on both *perceptive modality* and *hand* and with *group* as a categorical predictor. The analysis revealed a significant effect of *group* (F_(5,42)_ = 7.799, p<0.001) and *perceptive modality* (F_(5,42)_ = 12.849, p<0.001). The mean algebraic errors in degrees for the SV and subjective body Z-axis judgments were more pronounced in the AN group and in the tactile modality (see Tables 2 and 3). However, the interactions between *group* and *perceptive modality*, between *hand* and *perceptive modality* and between *hand*, *perceptive modality* and *group* were not statistically significant (n.s., p>0.25).

### Correlations with bodyweight and eating habits

To evaluate the effect of nutritional status (bodyweight) and eating disorders (the overall EDI-2 score and its “interoceptive awareness” subscore) on spatial cognition, correlation analyses of the study population as a whole and the AN group in particular were performed using Spearman's coefficient (ρ). The results are summarized in Table 4. Firstly, we found a significant, negative correlation between weight and spatial orientation judgments (SV or body midline) in both sensory modalities (visual and tactile) but only when the body was tilted. For tactile conditions, the analysis revealed a significant, negative correlation (i) between weight and tactile SV perception in the right-tilted position (ρ = −0.573, t_48 = _−4.843, p<0.001) and the left-tilted position (ρ = −0.452, t_48 = _−3.515, p<0.001) and (ii) between weight and tactile subjective body Z-axis perception in the right-tilted position (ρ = −0.515, t_48 = _−4.166, p<0.001) and in the left-tilted position (ρ = −0.343, t_48 = _−2.531, p = 0.014). For visual conditions, the analysis revealed a significant, negative correlation between weight and visual SV perception in the right-tilted position (ρ = −0.556, t_48 = _−4.631, p<0.001) and the left-tilted position (ρ = −0.449, t_48 = _−3.489, p = 0.001). A significant, negative correlation was also observed between weight and visual subjective body Z-axis perception in the right-tilted position (ρ = −0.508, t_48 = _−4.09, p<0.001) and the left-tilted position (ρ = −0.309, t_48 = _−2.25, p = 0.029). However, within the AN group, there were (i) significant negative correlations between weight and visual orientation judgments only when de body was upright (SV: ρ = −0.446, t_23 = _−2.39, p = 0.025; Z-axis: ρ = −0.411, t_23 = _−2.164, p = 0.04) and (ii) significant positive correlation between weight and tactile SV perception in the upright position (ρ = 0.408, t_48_ = 2.146, p = 0.043). Within the control group, there were no significant correlations between weight and spatial orientation judgments (all p>0.1, n.s.).

**Table 4:** Correlation analyses (r; Spearman tests) evaluating the effect of nutritional status and eating disorders on spatial cognition.

	Subjective vertical						Z-axis					
	Visual condition			Tactile condition			Visual condition			Tactile condition		
	Upright	CW	CCW	Upright	CW	CCW	Upright	CW	CCW	Upright	CW	CCW
Weight												
Total sample	−0.209	−0.609 ^a^	−0.564 ^a^	−0.122	−0.556 *^a^*	−0.449 *^b^*	−0.222	−0.508 ^a^	−0.309 ^c^	−0.109	−0.515 ^a^	−0.343 ^c^
AN group	−0.446 ^c^	−0.37 ^d^	−0.06	0.408 ^c^	−0.028	−0.032	−0.411 ^c^	−0.35 ^d^	−0.137	0.323	−0.226	−0.181
Control group	−0.232	−0.189	−0.329	−0.328	0.478 ^c^	−0.255	0.191	−0.139	0.199	−0.409 ^d^	−0.135	−0.182
EDI 2 total score												
Total sample	0.179	0.568 ^a^	0.325 ^c^	0.095	0.472 ^b^	0.488 *^a^*	0.138	0.44 ^b^	0.312 ^c^	0.059	0.418 ^b^	0.359 ^c^
AN group	0.095	0.183	0.116	0.279	0.065	0.066	0.096	0.09	0.179	0.279	0.006	0.247
Control group	0.042	0.144	0.017	0.072	0.056	0.329	0.116	0.129	0.264	0.024	0.07	0.06
EDI 2 IA												
Total sample	0.336 ^c^	0.593 ^a^	0.419 ^b^	0.078	0.47 ^b^	0.413 ^b^	0.326 ^c^	0.526 ^a^	0.339 ^c^	0.067	0.426 ^b^	0.315 ^c^
AN group	0.402 ^d^	0.16	0.122	0.208	0.137	0.141	0.402	0.02	0.049	0.28	0.294	0.091
Control group	0.178	0.346	0.423 ^c^	0.113	0.169	0.244	0.198	0.258	0.352 ^d^	0.147	0.285	0.138

*Significant differences (Spearman tests; two-tailed) are indicated: ^a^ p<0.001; ^b^ p<0.01; ^c^ p<0.05; ^d^ p < 0.1. CW: clockwise body tilted; CCW: counter-clockwise body tilted*.

In terms of eating habits, we observed significant, positive correlations between the EDI-2 score (the overall score and the “interoceptive awareness” subscore) and spatial orientation judgments (SV or subjective body Z-axis) for the study population as a whole and in both sensory modalities. The correlations were significant when the body was tilted. Under tactile conditions, the analysis revealed a significant, positive correlation (i) between the overall EDI-2 score and tactile SV perception in the right-tilted position (ρ = 0.472, t_42_ = 3.472, p = 0.001) and the left-tilted position (ρ = 0.488, t_42_ = 3.627, p<0.001) and (ii) between the “interoceptive awareness” subscore and tactile SV perception in the right-tilted position (ρ = 0.47, t_42_ = 3.472, p = 0.001) and in the left-tilted position (ρ = 0.413, t_42_ = 2.943, p = 0.005). Similar results were observed for subjective body Z-axis, with significant, positive correlations (i) between the overall EDI-2 score and tactile subjective body Z-axis perception in the right-tilted position (ρ = 0.418, t_42_ = 2.985, p = 0.005) and in the left-tilted position (ρ = 0.359, t_42_ = 2.499, p = 0.016) and (ii) between the “interoceptive awareness” subscore and tactile subjective body Z-axis perception in the right-tilted position (ρ = 0.426, t_42_ = 3.052, p = 0.004) and in the left-tilted position (ρ = 0.315, t_42_ = 2.151, p = 0.037). Under visual conditions, the analysis revealed a significant, positive correlation (i) between the overall EDI-2 score and visual SV perception in the right-tilted position (ρ = 0.568, t_42_ = 4.469, p<0.001) and in the left-tilted position (ρ = 0.325, t_42_ = 2.226, p = 0.031) (ii) between the “interoceptive awareness” subscore and visual SV perception in the upright position (ρ = 0.336, t_42_ = 2.31, p = 0.026), the right-tilted position (ρ = 0.593, t_42_ = 4.777, p<0.001) and the left-tilted position (ρ = 0.419, t_42_ = 2.993, p = 0.005). Similar results were observed for subjective body Z-axis, with significant, positive correlations (i) between the overall EDI-2 score and visual subjective body Z-axis perception in the right-tilted position (ρ = 0.44, t_42_ = 3.18, p = 0.003) and in the left-tilted position (ρ = 0.312, t_42_ = 2.13, p = 0.039) and (ii) between the “interoceptive awareness” subscore and visual subjective body Z-axis perception in the upright position (ρ = 0.326, t_42_ = 2.21, p = 0.033), the right-tilted position (ρ = 0.526, t_42_ = 4.012, p<0.001) and the left-tilted position (ρ = 0.339, t_42_ = 2.342, p = 0.024).

An ANCOVA was used to identify which of the individual parameters (body mass index, overall EDI-2 score, “interoceptive awareness” EDI-2 subscore and group) played a dominant role on the spatial orientation judgments (SV or body midline) in both sensory modalities (visual and tactile). The results are summarized in Table 5. Group was positively associated with (i) tactile SV and Z-axis perception in the right-tilted position (F_SV(1,46)_ = 6.135, p = 0.018; F_Z(1,46)_ = 2.593, p = 0.05), and with (ii) visual SV perception in the right-tilted position (F_(1,46)_ = 6.751, p = 0.013). Body mass index was positively associated with visual SV and Z-axis perception in left-tilted position (F_SV(1,46)_ = 5.589, p = 0.023; F_Z(1,46)_ = 5.445, p = 0.025). Finally, visual VS and Z-axis perception in the upright position were positively associated with the “interoceptive awareness” subscore (F_(1,46)_ = 2.554, p = 0.05).

**Table 5:** Results of the ANCOVA (F) exploring the influence of physiological and psychological measures on spatial tasks.

	TU	TSVCW	TSVCCW	TZCW	TZCCW	VU	VSVCW	VSVCCW	VZCW	VZCCW
BMI	0.891	1.474	0.474	0.083	0.565	0.036	0.108	5.589^ a^	1.598^ b^	5.445^ a^
EDI-2 TS	0.634	0.498	0.596	0.031	0.943	1.255	0.102	0.027	0.129	0.272
EDI-2 IA	0.043	0.77	0.128	0.222	0.364	2.554^ a^	0.730	0.108	0.251	0.675
Group	0.115	6.135^ a^	2.218^ b^	2.593^ a^	2.082^ b^	0.001	6.751^ a^	0.048	1.397	0.114

*Significant differences (analysis of covariance; one-tailed) are indicated: ^a^ p<0.05; ^b^ p<0.1. TU: tactile up-right body; TSVCW: tactile subjective vertical clockwise body tilted; TSVCCW: tactile subjective vertical counter-clockwise body tilted; TZCW: tactile Z-axis clockwise body tilted; TZCCW: tactile Z-axis counter-clockwise body tilted; VU: visual up-right body; VSVCW: visual subjective vertical clockwise body tilted; VSVCCW: visual subjective vertical counter-clockwise body tilted; VZCW: visual Z-axis clockwise body tilted; VZCCW: visual Z-axis counter-clockwise body tilted; BMI: body mass index; EDI-2 TS: eating disorder inventory 2 total score; EDI-2 IA: eating disorder inventory 2 interoceptive awareness*.

## Discussion

The aim of the present study was to investigate spatial cognition in AN, as evidenced by spatial orientation constancy and perception of the SV and body posture in two different sensory modalities. Spatial cognition was analyzed in patients with AN and healthy control participant by using tasks involving the manual adjustment of a rod to the vertical or the body midline when the body was upright and when it was tilted. In order to establish whether AN patients have direction-specific impairments in tactile-spatial and visual-spatial orientation, we applied tasks involving the integration of visual, vestibular and proprioceptive information [29], [31]–[33]. Furthermore, we looked at whether impaired performance in axis orientation tasks was modulated by the use of an egocentric reference frame vs. an allocentric reference frame.

### Control tasks

First, study participants performed control tasks, in order to confirm the absence of impairments in unimodal perception. Our results showed that the AN and control groups had similar performance levels when discriminating between simple-shaped objects (Experiment 1 & 2) in both visual and tactile modalities. With respect to tactile exploration, our results contrast to some extent with those in the literature research [10], [41] because we did not observe the impairment of tactile exploration in AN patients. This divergence may be due to a difference in the complexity of the respective tasks. In Grunwald *et al.* 's experiment [10], the tactile exploration tasks consisted of sequentially palpating the structure of six sunken reliefs with both hands and the eyes closed. After each palpation, the structure had to be copied on a piece of paper. The quality of reproduction of complex stimuli was notably lower for AN patients than for healthy controls, regardless of the weight gain. In the haptic illusion task developed by Tchanturia *et al.* [41], participants are asked to judge the relative size of wooden balls rolled into each hand whilst their eyes were closed. After a habituation phase involving two balls of different sizes (15 trials), two same-sized balls are presented. Anorexic patients experienced a greater number of perceptual illusions (i.e. same-sized balls were perceived as being of different sizes) than control participants did [45]. However, this experimental task does not distinguish between impaired tactile exploration (i.e. a decrease in the ability to discriminate the objects' size) and impaired set-shifting ability (i.e. a decrease of the cognitive flexibility) [20], [41], [45]. Hence, these literature results do not necessarily suggest that tactile size discrimination is impaired in AN. With regard to visual discrimination, we found that AN patients and healthy controls had similar performance levels. The present results confirm previous work in which the visual modality was found not to be affected in AN [7] and suggest that unimodal visual or tactile perception is not impaired in this disease – at least in terms of the discrimination of simple shapes.

### The subjective vertical

In both groups, the tactile and visual SV measured in the upright position was very close to the gravitational vertical. However, tilting the body led to significant deviations of the tactile and visual SV. In contrast to the E-effect found in previous studies when the SV was estimated with tactile adjustments only [31], [32], [35], [36], we observed a small but statistically significant A-effect in the control group. This could be due to methodological factors, such as a gender effect [34]. It is noteworthy that only young women participated in our experiment; this contrasts with previous studies, in which most of the participants were male. Other methodological factors (such as the length of the adjustable bar or the use of a tilted chair vs. the body lying on a tilted surface) may also be involved in this difference.

As reported previously [37], our results indicate that the A-effect yielded by a body tilt was abnormally high in the AN patients, whereas no difference between the AN and control groups was observed when the participants were in an upright position. Furthermore, the impairment in spatial orientation was observed in both tactile and visual modalities. Moreover, the AN patients' impairment was not due to less precise adjustments, since there was no significant intergroup difference in the individual SD (i.e. the intra-individual variability of adjustment). Thus, our results suggest a shift in the distribution of the AN population's A-effect (i.e. a specific source of bias) and thus the presence of a supramodal impairment in spatial orientation constancy in AN. Our results reinforce the hypothesis whereby the PC has a key role in AN [10], [11], [13], [39]. Indeed, Pérennou *et al.* [46] recently showed that the most marked visual and tactile tilts in the frontal plane were associated with right parietal lesions, suggesting that an internal model of verticality is elaborated in the right PC. Reports of multimodal neurons [47], [48] and ‘axis-orientation-selective’ [49] neurons in the PC further suggest that the PC is the anatomical substrate of a supramodal spatial reference frame [29]. According to Funk *et al.* [29], the PC “has been shown to receive visual signals and eye-position signals, as well as efference copies of motor signals, vestibular signals and neck proprioceptive signals to account for head orientation and head movements in space. Damage to the right posterior PC might therefore lead to a systematic error in the integration of information – as for example somatosensory (head-position) and graviceptive (vestibular) input – in neglect patients”. In AN, dysfunction of this type of network would disturb the representation of space. We have previously suggested that the A-effect may mean that the tilted individual can only access a subjective gravitational frame of reference (biased towards the direction of the body's orientation in space), rather than an objective frame of reference [32]. Thus, the abnormally elevated A-effect in anorexics could be interpreted as greater dependence on the egocentric frame of reference as a result of failure to accurately integrate gravitational cues (such as vestibular information) in the PC [37]. To investigate this hypothesis, we therefore asked participants to adjust a phosphorescent line to the body midline under a visual condition and to adjust a rod to the body midline under a tactile condition. Since this task would involve the egocentric frame of reference more directly, we expected the AN patients to be able to judge their Z-axis position in space with greater accuracy (relative to their performance in the SV task).

### The body Z-axis task

In fact, our results disproved the above-mentioned hypothesis. Tactile and visual body Z-axis judgments in the upright position were similar in the two groups. However, tilting led to significant deviations of the tactile and visual body Z-axis, with participants judging that the body was more tilted than it really was. In the control and AN groups, this result was in line with previous data showing that when tilted participants were asked to adjust a visual rod parallel to their body axis in a dark environment, a deviation of the rod towards the direction of tilt was observed [50], [51]. In the AN group, we observed a more pronounced bias toward the tilt. This pattern of results in AN patients cannot be explained by higher weighting with respect to the egocentric frame of reference (also referred to as “the idiotropic vector”) [38]. A second influential model of the perception of space suggests that judgements could reflect gravitational inflow, such as changes in vestibular, kinaesthetic and somesthetic inputs [30], [52]. Several studies have shown that somatosensory information is involved in the A-effect [52], [53]. Likewise, a patient with hemiparesis was found to have impaired judgment of verticality when he was leaning to the hemiparetic side [54]. Thus, the forces exerted by body mass may contribute to somatic graviception [55].

### Correlations between spatial task performance and somatosensory information

Interestingly, the present study evidenced a negative correlation between the constant errors in degrees in tactile-spatial orientation tasks and body weight for the study population as a whole. However, this was not the case in the AN group. The analysis of covariance used to distinguish the influence of weight and group showed a stronger involvement of the group effect in tactile modality. However, the weight could have a smaller effect in visual modality, particularly when the body is tilted. These results are partially consistent with several literature studies. Subjects with the highest bodyweight undergo a stronger gravitational force. Thus, they may receive more somatosensory information and be more accurate in their determination of the SV and the body Z-axis [55]. However, in a disease setting, gravitational inflow may not be strong enough to maintain a constant frame of reference. We found a negative relationship between interoceptive awareness and the A-effect; the lower the interoceptive awareness, the greater the A-effect. Impaired consideration of body signal may participate to the disruption of spatial cognition. Indeed, patients with AN suffer from a lack of interoceptive awareness and perceive bodily signals less intensely [21]. This observation prompts us to suggest that poor perception of orientation could be related to poor awareness of interoceptive signals. As recently suggested by Barra et al. [56], the awareness of body orientation could modulate verticality representation. In addition to sensory integration, attentional processes might play also a role in the sense of verticality. This suggested that both bottom-up and top-down processes would be involved in AN. Taken as a whole, our results support the hypothesis whereby spatial cognition is impaired in AN. The disruption of spatial references may be involved in the development of a distorted dynamic sensorimotor representation of the body. In turn, this would worsen the prognosis in AN by increasing body dissatisfaction and the obsessive will to lose weight and thus maintaining restrictive eating behaviours [57]–[60].

Nevertheless, our study had a number of limitations. Firstly, an ANOVA of the data obtained under visual judgment conditions revealed not only an effect of *body orientation* and an interaction between *body orientation* and *group* but also an effect of *hand* and an interaction between *hand* and *body orientation*. Performance was best with the hand opposite to the direction of body tilt. This may have been a hardware limitation, since pressure on the arm under the tilted body would have made the task more difficult. It would be useful to duplicate the present study using a tiltable seat. Secondly, our study did not provide direct evidence of an association between AN and parietal dysfunction. In fact, we used techniques and a conceptual framework that had been used successfully in neuroscience research with patients suffering from parietal lesions [29], [46]. Further experiments are needed, e.g. a comparison between a wider range of populations (healthy participants, AN patients and hemineglect and psychiatric patients unaffected by AN). Finally, regarding to the influence of physiological and psychological measures on spatial tasks, loss of significance in the subgroup analysis could be due to a lack of statistical power. Future studies should focus on larger cohorts.

In conclusion, our present findings evidenced impaired spatial task performance in AN; this may have been due to impaired integration of visual, tactile and gravitational information (e.g. vestibular and proprioceptive cues) in the PC. One can legitimately hypothesize that PC dysfunction disturbs the integration of the body-orientation representation needed to achieve spatial orientation constancy. Our study results also suggest that special attention should be given to neuropsychology in the diagnostic management and treatment of AN [13]. There is a need for further research on the impairments observed here; with a focus on the influence of altered spatial references on social frameworks and the implications for social cognition [61]–[63]. Even if conflicting results exist concerning a possible effect of AN on social cognition [64], several studies have shown that emotional theory of mind and empathy could be altered in AN [65]–[67]. Our clinical experience leads us to question the ability of patients with AN to infer mental states other than their own. A better understanding of the social difficulties encountered by patients would enable the development of more appropriate cognitive remediation therapies. Spatial cognition in patients with AN may be a promising initial therapeutic target.

## References

[1] American Psychiatric Association (2000) The diagnostic and statistical manual of mental disorders – Text revision (4th ed. Text revised). Washington, DC: Author.

[2] De Vignemont F (2010) Body schema and body image–pros and cons. Neuropsychologia 48: 669–680. 19786038 10.1016/j.neuropsychologia.2009.09.022

[3] Gallagher S (2005) How the body shapes the mind. New York: Oxford University Press.

[4] Paillard J (1999) Body schema and body image: A double dissociation in deafferented patients G.N. Gantchev, S. Mori, J. Massion (Eds.), Motor control, today and tomorrow, 197–214

[5] Schwoebel J, Coslett HB (2005) Evidence for multiple, distinct representations of the human body. J Cogn Neurosci 17: 543–553. 15829076 10.1162/0898929053467587

[6] Cash TE, Deagle EA (1997) The nature and extent of body-image disturbances in anorexia nervosa and bulimia nervosa: A meta-analysis. Int J Eat Disord 22: 107–125. 9261648

[7] Smeets MA, Ingleby JD, Hoek HW, Panhuysen GE (1999) Body size perception in anorexia nervosa: a signal detection approach. J Psychosom Res 46: 465–477. 10404481 10.1016/s0022-3999(99)00005-7

[8] Skrzypek S, Wehmeier PM, Remschmidt H (2001) Body image assessment using body size estimation in recent studies on anorexia nervosa. A brief review. Eur Child Adolesc Psychiatry 10: 215–221. 11794546 10.1007/s007870170010

[9] Farrell C, Lee M, Shafran R (2005) Assessment of body size estimation: A review. Eur Eat Disord Rev 13: 75–88.

[10] Grunwald M, Ettrich C, Krause W, Assmann B, Dähne A, et al (2001) Haptic perception in anorexia nervosa before and after weight gain. J Clin Exp Neuropsychol 23: 520–529. 11780950 10.1076/jcen.23.4.520.1229

[11] Wagner A, Ruf M, Braus DF, Schmidt MH (2003) Neuronal activity changes and body image distortion in anorexia nervosa. NeuroReport 14: 2193–2197. 14625446 10.1097/00001756-200312020-00012

[12] Guardia D, Lafargue G, Thomas P, Dodin V, Cottencin O, et al (2010) Anticipation of body-scaled action is modified in anorexia nervosa. Neuropsychologia 48: 3961–3966. 20833193 10.1016/j.neuropsychologia.2010.09.004

[13] Nico D, Daprati E, Nighoghossian N, Carrier E, Duhamel JR, et al (2010) The role of the right parietal lobe in anorexia nervosa. Psychol Med 40: 1531–1539. 19917144 10.1017/S0033291709991851

[14] Guardia D, Conversy L, Jardri R, Lafargue G, Thomas P, et al.. (2012) Imagining one's own and someone else's body actions: a dissociation in anorexia nervosa. Plos One (in press). 10.1371/journal.pone.0043241 PMC3425562 22937025

[15] Tomasino SJ (1996) Does right parietal cortex and vestibular dysfunction underlie body image distortion? J Nerv Ment Dis 184: 758. 8994460 10.1097/00005053-199612000-00007

[16] Grunwald M, Ettrich C, Busse F, Assmann B, Dähne A, et al (2002) Angle paradigm: A new method to measure right parietal dysfunctions in anorexia nervosa. Arch Clin Neuropsychol 17: 485–496. 14592002

[17] Daprati E, Sirigu A, Nico D (2010) Body and movement: consciousness in the parietal lobes. Neuropsychologia 48: 756–762. 19837100 10.1016/j.neuropsychologia.2009.10.008

[18] Tchanturia K, Serpell L, Troop N, Treasure J (2001) Perceptual illusions in eating disorders: rigid and fluctuating styles. J Behav Ther Exp Psychiatry 32: 107–115. 11934124 10.1016/s0005-7916(01)00025-8

[19] Grunwald M, Weiss T, Assmann B, Ettrich C (2004) Stable asymmetric interhemispheric theta power in patients with anorexia nervosa during haptic perception even after weight gain: a longitudinal study. J Clin Exp Neuropsychol 26: 608–620. 15370383 10.1080/13803390409609785

[20] Roberts ME, Tchanturia K, Stahl D, Southgate L, Treasure J (2007) A systematic review and meta-analysis of set-shifting ability in eating disorders. Psychol Med 37: 1075–1084. 17261218 10.1017/S0033291707009877

[21] Pollatos O, Kurz AL, Albrecht J, Schreder T, Kleemann AM, et al (2008) Reduced perception of bodily signals in anorexia nervosa. Eat Behav 9: 381–388. 18928900 10.1016/j.eatbeh.2008.02.001

[22] Keizer A, Aldegonda M, Smeets M, Dijkerman H, Postma A (2011) Tactile body image disturbance in anorexia nervosa. Psychiatry Res 190: 115–120. 21621275 10.1016/j.psychres.2011.04.031

[23] Case LK, Wilson RC, Ramachandran VS (2012) Diminished size-weight illusion in anorexia nervosa: evidence for visuo-proprioceptive integration deficit. Exp Brain Res 217: 79–87. 22183754 10.1007/s00221-011-2974-7

[24] Eshkevari E, Rieger E, Longo MR, Haggard P, Treasure J (2012) Increased plasticity of the bodily self in eating disorders. Psychol Med 42: 819–828. 22017964 10.1017/S0033291711002091

[25] Charpentier A (1891) Analyse experimentale: De quelques elements de la sensation de poids. Archives de Physiologie Normale et Pathologique 3: 122–135.

[26] Botvinick M, Cohen J (1998) Rubber hands ‘feel’ touch that eyes see. Nature 391: 756. 9486643 10.1038/35784

[27] Colby CL, Duhamel JR (1996) Spatial representations for action in parietal cortex. Cog Brain Res 5: 105–115. 10.1016/s0926-6410(96)00046-8 9049076

[28] Kerkhoff G (1999) Multimodal spatial orientation deficits in left-sided visual neglect. Neuropsychologia 37: 1387–1405. 10606013 10.1016/s0028-3932(99)00031-7

[29] Funk J, Finke K, Müller HJ, Utz KS, Kerkhoff G (2010) Effects of lateral head inclination on multimodal spatial orientation judgments in neglect: evidence for impaired spatial orientation constancy. Neuropsychologia 48: 1616–1627. 20138897 10.1016/j.neuropsychologia.2010.01.029

[30] Howard I (1982) Human Visual Orientation. Wiley, New York.

[31] Gentaz E, Baud-Bovy G, Luyat M (2008) The haptic perception of spatial orientations. Exp Brain Res 187: 331–348. 18446332 10.1007/s00221-008-1382-0 PMC2373857

[32] Luyat M, Gentaz E, Corte TR, Guerraz M (2001) Reference frames and haptic perception of orientation: body and head tilt effects on the oblique effect. Percept Psychophys 63: 541–554. 11414140 10.3758/BF03194419

[33] Luyat M, Gentaz E (2002) Body tilt effect on the reproduction of orientations: studies on the visual oblique effect and subjective orientations. J Exp Psychol Hum Percept Perform 28: 1002–1011. 12190248

[34] Luyat M, Noel M, Thery V, Gentaz E (2012) Gender and line size factors modulate the deviations of the subjective visual vertical induced by head tilt. BMC Neurosci 13: 28. 22420467 10.1186/1471-2202-13-28 PMC3329413

[35] Bauermeister M, Werner H, Wapner S (1964) The effect of body tilt on tactual-kinesthetic perception of verticality. Am J Psychol 77: 451–456. 14198668

[36] Bortolami SB, Pierobon A, DiZio P, Lackner JR (2006) Localization of the subjective vertical during roll, pitch, and recumbent yaw body tilt. Exp Brain Res 173: 364–373. 16628401 10.1007/s00221-006-0385-y

[37] Guardia D, Cottencin O, Thomas P, Dodin V, Luyat M (2012) Spatial orientation constancy is impaired in anorexia nervosa. Psychiatry Res 195: 56–59. 21872340 10.1016/j.psychres.2011.08.003

[38] Mittelstaedt H (1983) A new solution to the problem of the subjective vertical. Naturwissenschaften 70: 272–281. 6877388 10.1007/BF00404833

[39] Kinsbourne M, Bemporad JR (1984) Lateralization of emotion: a model and the evidence. In The psychobiology of affective development (ed. A Fox, RJ Davidson), 259–291. Lawrence Erlbaum: Hillsdale, NJ.

[40] Ceyte H, Cian C, Trousselard M, Barraud PA (2009) Influence of perceived egocentric coordinates on the subjective visual vertical. Neurosci Lett 462: 85–88. 19545600 10.1016/j.neulet.2009.06.048

[41] Tchanturia K, Anderluh MB, Morris RG, Rabe-Hesketh S, Collier DA, et al (2004) Cognitive flexibility in anorexia nervosa and bulimia nervosa. J Int Neuropsychol Soc 10: 513–520. 15327730 10.1017/S1355617704104086

[42] Sheehan DV, Lecrubier Y, Sheehan KH, Amorim P, Janavs J, et al (1998) The mini-international neuropsychiatric interview (MINI): The development and validation of a structured diagnostic psychiatric interview for DSM-IV and ICD-10. J Clin Psychiatry 59: 22–33. 9881538

[43] Oldfield RC (1971) The assessment and analysis of handedness: the Edinburgh inventory. Neuropsychologia 9: 97–113. 5146491 10.1016/0028-3932(71)90067-4

[44] Garner DM (1991) EDI 2: Eating Disorder Inventory 2. In Professional Manual. Psychology Assessment Resources Inc, Odessa, FL, USA.

[45] Roberts ME, Tchanturia K, Treasure JL (2010) Exploring the neurocognitive signature of poor set-shifting in anorexia and bulimia nervosa. J Psychiatry Res 44: 964–970. 10.1016/j.jpsychires.2010.03.001 20398910

[46] Pérennou D, Mazibrada G, Chauvineau V, Greenwood R, Rothwell J, et al (2008) Lateropulsion, pushing and verticality perception in hemispheric stroke: A causal relationship? Brain 131: 2401–2413. 18678565 10.1093/brain/awn170

[47] Duhamel JR, Colby CL, Goldberg ME (1998) Ventral intra-parietal area of the macaque: Congruent visual and somatic response properties. J Neurophysiol 79: 126–136. 9425183 10.1152/jn.1998.79.1.126

[48] Graziano MSA, Gross CG (1995) The representation of extrapersonal space: A possible role for bimodal, visual-tactile neurons. In M. S. Gazzaniga (Ed.), The Cognitive Neurosciences (1021–1034). Cambridge, MA: The MIT Press.

[49] Sakata H, Taira M, Kusunoki M, Murata A, Tanaka Y (1997) The parietal association cortex in depth perception and visual control of hand action. Trends Neurosci 20: 350–357. 9246729 10.1016/s0166-2236(97)01067-9

[50] Bauermeister M (1964) Effect of body tilt on apparent verticality, apparent body position, and their relation. J Exp Psychol 67: 142–147. 14114911 10.1037/h0046424

[51] Ceyte H, Cian C, Nougier V, Olivier I, Trousselard M (2007) Role of gravity-based information on the orientation and localization of the perceived body midline. Exp Brain Res 176: 504–509. 17180705 10.1007/s00221-006-0764-4

[52] Trousselard M, Barraud P, Nougier V, Raphel C, Cian C (2004) Contribution of tactile and interoceptive cues to the perception of the direction of gravity. Brain Res Cog Brain Res 20: 355–362. 10.1016/j.cogbrainres.2004.03.008 15268913

[53] Mittelstaedt H (1997) Interaction of eye-, head-, and trunk-bound information in spatial perception and control. J Vestib Res 7: 283–302. 9218243

[54] Anastasopoulos D, Bronstein A, Haslwanter T, Fetter M, Dichgans J (1999) The role of somatosensory input for the perception of verticality. Ann N Y Acad Sci 871: 379–383. 10372086 10.1111/j.1749-6632.1999.tb09199.x

[55] Mittelstaedt H (1996) Somatic graviception. Biol Psychol 42: 53–74. 8770370 10.1016/0301-0511(95)05146-5

[56] Barra J, Pérennou D, Thilo KV, Gresty MA, Bronstein AM (2012) The awareness of body orientation modulates the perception of visual vertical. Neuropsychologia 50(10), 2492–2498. 10.1016/j.neuropsychologia.2012.06.021 22766439

[57] Exterkate CC, Vriesendorp PF, De Jong CAJ (2009) Body attitudes in patients with eating disorders at presentation and completion of intensive outpatient day treatment. Eat Behav 10: 16–21. 19171312 10.1016/j.eatbeh.2008.10.002

[58] Santonastaso P, Bosello R, Schiavone P, Tenconi E, Degortes D, et al (2009) Typical and atypical restrictive anorexia nervosa: weight history, body image, psychiatric symptoms, and response to outpatient treatment. Int J Eat Disord 42: 464–470. 19424978 10.1002/eat.20706

[59] Franko DL, Striegel-Moore RH (2002) The role of body dissatisfaction as a risk factor for depression in adolescent girls: Are the differences black and white? J Psychosom Res 53: 975–983. 12445587 10.1016/s0022-3999(02)00490-7

[60] Heilbrun AB, Friedberg L (1990) Distorted body image in normal college women: Possible implications for the development of anorexia nervosa. J Clin Psychol 46: 398–401. 2212040 10.1002/1097-4679(199007)46:4<398::aid-jclp2270460404>3.0.co;2-5

[61] Nico D, Daprati E (2009) The egocentric reference for visual exploration and orientation. Brain Cog 69: 227–235. 10.1016/j.bandc.2008.07.011 18752882

[62] De Vignemont F (2008) Frames of reference in social cognition. Quarterly Journal of Experimental Psychology 61, 90–100. 10.1080/17470210701508764 18038341

[63] Frith U, De Vignemont F (2005) Egocentrism, allocentrism, and Asperger syndrome. Conscious Cog 14: 719–738. 10.1016/j.concog.2005.04.006 15996486

[64] Adenzato M, Todisco P, Ardito RB (2012) Social cognition in anorexia nervosa: evidence of preserved theory of mind and impaired emotional functioning. PLoS One 7(8), e44414. 10.1371/journal.pone.0044414 PMC3432108 22952975

[65] McAdams CJ, Krawczyk DC (2011) Impaired neural processing of social attribution in anorexia nervosa. Psychiatry Res 194: 54–63. 21872451 10.1016/j.pscychresns.2011.06.016

[66] Oldershaw A, Hambrook D, Tchanturia K, Treasure J, Schmidt U (2010) Emotional theory of mind and emotional awareness in recovered anorexia nervosa patients. Psychosom Med 72: 73–79. 19995886 10.1097/PSY.0b013e3181c6c7ca

[67] Russell TA, Schmidt U, Doherty L, Young V, Tchanturia K (2009) Aspects of social cognition in anorexia nervosa: affective and cognitive theory of mind. Psychiatry Res 168: 181–185. 19467562 10.1016/j.psychres.2008.10.028

